# The rise of viperin: the emerging role of viperin in cancer progression

**DOI:** 10.1172/JCI165907

**Published:** 2022-12-15

**Authors:** Alyssa G. Weinstein, Inês Godet, Daniele M. Gilkes

**Affiliations:** 1 Department of Oncology, The Sidney Kimmel Comprehensive Cancer Center and; 2 Biochemistry and Molecular Biology Program, The Johns Hopkins University School of Public Health, Baltimore, Maryland, USA.; 3 Department of Chemical and Biomolecular Engineering and; 4 Johns Hopkins Institute for NanoBioTechnology, The Johns Hopkins University, Baltimore, Maryland, USA.; 5 Cellular and Molecular Medicine Program, The Johns Hopkins University School of Medicine, Baltimore, Maryland, USA.

## Abstract

Viperin, an IFN-regulated gene product, is known to inhibit fatty acid β-oxidation in the mitochondria, which enhances glycolysis and lipogenesis during viral infections. Yet, its role in altering the phenotype of cancer cells has not been established. In this issue of the *JCI*, Choi, Kim, and co-authors report on a role of viperin in regulating metabolic alterations in cancer cells. The authors showed a correlation between clinical outcomes and viperin expression levels in multiple cancer tissues and proposed that viperin expression was upregulated in the tumor microenvironment via the JAK/STAT and PI3K/AKT/mTOR/HIF-1α pathways. Functionally, viperin increased lipogenesis and glycolysis in cancer cells by inhibiting fatty acid β-oxidation. Viperin expression also enhanced cancer stem cell properties, ultimately promoting tumor initiation in murine models. This study proposes a protumorigenic role for viperin and identifies HIF-1α as a transcription factor that increases viperin expression under serum starvation and hypoxia.

## Metabolic reprogramming makes cancer cells more lethal

Metabolic reprogramming is a hallmark of malignancy that allows cancer cells to utilize nutrients and energy to continuously proliferate (1). Proliferating cancer cells have increased rates of glycolysis and macromolecule biosynthesis. While normal cells require extracellular signals to proliferate, cancer cells have metabolic autonomy and do not always need extracellular signals to trigger proliferation (2). The most well-understood metabolic changes that occur during cancer progression include alterations in the PI3K/AKT/mTOR pathway, stabilization of HIF-1α, and enhanced expression of *MYC* genes (3–5). Recent data support the role of IFNs in regulating cancer metabolism; for example, IFNs can activate the JAK/STAT signaling pathway in cancer cells to regulate metabolic processes and activate a tumor immune response (6). However, the role of IFN-stimulated genes (ISGs) in metabolic reprogramming is not fully understood. In this issue of the *JCI*, Choi, Kim, and co-authors addressed this knowledge gap by examining the role of the IFN-inducible protein viperin in metabolic reprogramming (7). Their data suggest that viperin is the ISG that controls cancer cell metabolism, whereas other ISGs that are upregulated in cancer cells may not alter metabolism.

## The emerging role of viperin in cancer

Viperin plays important roles in several cell types, including fibroblasts, adipocytes, and macrophages. It interacts with proteins to inhibit fatty acid β-oxidation in the mitochondria of fibroblasts and adipocytes (8, 9). Viperin also inhibits viral replication and activates IFN expression in macrophages (10). Until recently, the role of viperin in cancer cells remained unclear. The study by Choi, Kim, and colleagues reported that viperin expression was increased in gastric, lung, and breast cancer cells compared with expression in normal cells, based on tissue microarray data. Immunohistochemistry showed that cytoplasmic viperin labeling varied in intensity across different tumors. Likewise, viperin was not ubiquitously overexpressed in every cancer cell line that the research group tested. However, using RNA expression data available in the The Cancer Genome Atlas (TCGA) database, the authors showed that viperin expression correlated with worse clinical outcomes in patients with gastric, lung, breast, pancreatic, kidney, and brain cancer (7).

## Viperin promotes metabolic reprogramming

One key clue indicating that viperin might play a role in metabolic reprogramming, aside from induction by IFN-γ, was viperin’s localization to the mitochondria in cancer cells. In addition to being the cell’s powerhouse, mitochondria are also responsible for generating the precursors necessary to form macromolecules such as lipids and proteins (11). Choi, Kim, and colleagues found that viperin-expressing cells accumulated lipid droplets (LDs). Depleting viperin reduced LD formation but enhanced fatty acid β-oxidation, thus confirming its role in promoting lipogenesis. Likewise, viperin also promoted glycolysis, as measured by a decreased extracellular acidification rate in viperin-knockdown cells. Viperin affected glycolysis by enhancing the expression of glucose transporters 1 and 4 (GLUT1/-4) as well as the sterol-regulatory (SR) and carbohydrate-responsive (ChR) element–binding proteins (EBPs). Overexpressing viperin-mutant proteins lacking either a mitochondrial localization sequence or an iron-sulfur (Fe-S) cluster–binding domain did not affect lipogenesis or glycolysis. The results demonstrated the requirement for both viperin mitochondrial localization and Fe-S binding for metabolic reprogramming. Interestingly, viperin expression levels did not affect lipogenesis in the absence of glucose. In vivo experiments supported the group’s in vitro findings. For example, tumors from mice bearing MKN28 viperin-knockdown cells had reduced levels of viperin, GLUT4, SREBP, and ChREBP compared with MKN28 control tumors (7).

## Factors that promote viperin expression

**Figure 1:**
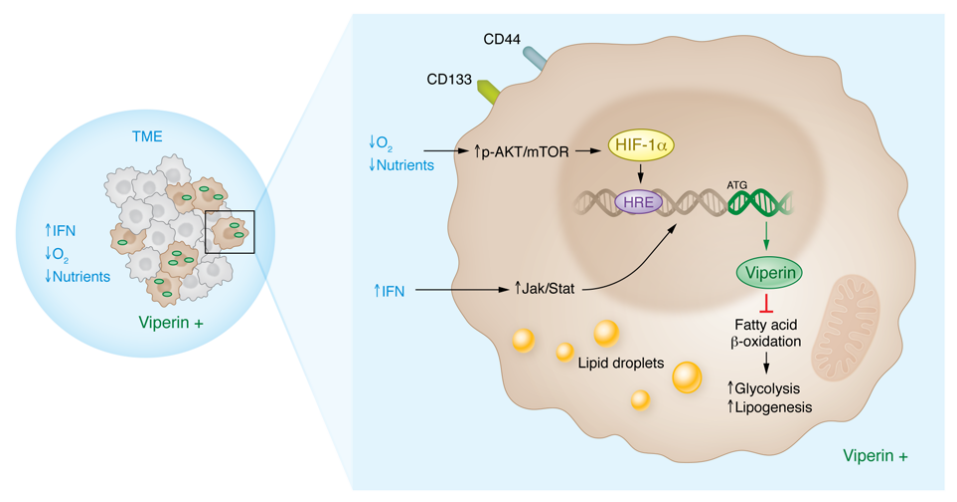
Viperin promotes cancer cell proliferation in low-oxygen and/or nutrient-deprived conditions in the TME. Choi, Kim, and colleagues report that cancer cells expressing high levels of viperin have increased levels of glycolysis and lipogenesis in the absence of oxygen and nutrients. This metabolic reprogramming occurs via two pathways: (a) the phosphorylated AKT/mTOR (p-AKT/mTOR) pathway, which is stimulated by a lack of oxygen and nutrients and activates downstream transcription factors, such as HIF1-α, to increase the transcription of viperin; and (b) the JAK/STAT pathway, which is activated by increased production of IFNs, where STAT binds directly to the DNA to increase viperin transcription. Viperin inhibits fatty acid β-oxidation, thus increasing glycolysis and lipogenesis in the presence of glucose. Additionally, viperin expression coincides with the enrichment of CSC properties (7).

Cancer cells must adapt to a hostile tumor microenvironment (TME) in order to survive (12). The TME has been characterized as being deprived of nutrients and oxygen while containing growth factors and cytokines that alter cancer cell behavior (13). To determine whether external factors in the TME could promote the expression of viperin, the research team exposed cells to IFN-γ, hypoxia, or serum starvation (7). Each condition independently induced the expression of viperin in MKN28 cells, with more substantial increases elicited by subjecting cells to IFN-γ or serum starvation compared with exposure to hypoxic conditions (7) (Figure 1).

Previous studies have shown that viperin is an IFN-γ–inducible gene product (14), but the mechanisms whereby hypoxia or serum starvation induced viperin expression had not yet been explored until now. Choi, Kim, and colleagues used chemical inhibitors and an siRNA-knockdown approach to determine that the PI3K/AKT/mTOR/HIF-1α signaling pathway induced viperin expression under both serum-starved and hypoxic conditions. In follow-on experiments, the group found that the viperin gene contained a hypoxia-responsive–binding element (HRE) within the 5′ promoter region. ChIP experiments revealed that HIF-1α was bound to this region upon serum starvation or exposure to hypoxia. Ultimately, viperin expression was required for serum starvation–, hypoxia-, and IFN-γ–triggered lipogenesis and glycolysis (7).

## Factors that restrict viperin expression

Many feedback systems in biology are negative feedback systems. Negative feedback is essential to limit a signaling response that has been activated but is no longer required (15). Two classic examples are pathways involving the p53 and HIF-1α proteins. Both proteins accumulate following stress, such as DNA damage or hypoxia, and then rapidly degrade, returning to basal levels when the stressor has been alleviated (16, 17). In a similar sense, viperin expression is induced under serum-starved conditions, and this induction can be reversed by adding serum or supplementing with DMEM/F12 or B27. More specifically, Choi, Kim, and co-authors discovered that the elements in common between serum and the media supplements were linoleic acid, a polyunsaturated fatty acid, and putrescine, a precursor of polyamine biosynthesis. The addition of linoleic acid, but not putrescine, suppressed viperin induction under serum-starved conditions. Likewise, oleic and palmitic fatty acids had an effect similar to that of adding linoleic acid to serum-free media. The addition of oleic acid also suppressed serum starvation–induced HIF-1α expression, suggesting that HIF-1α serves as the negative feedback switch that controls viperin expression (7).

## Viperin and the cancer stem cell phenotype

Tumor heterogeneity is caused by genetic and epigenetic alterations and the existence of cancer stem cells (CSCs). CSCs are undifferentiated tumor cells with self-renewal properties that promote tumor formation and cause resistance to therapy (18). CSCs have a tendency to localize within hypoxic tumor regions to preserve stemness and tumorigenic properties (19). Hypoxia-inducible factors (HIFs), particularly HIF-1α, drive enhancement and maintenance of stem-like properties by regulating transcripts associated with pluripotency, glycolysis, and drug resistance (20, 21). Choi, Kim, and colleagues reported that viperin was present in the small population of HIF-1α–expressing cells under both standard and serum-starved conditions, suggesting that these cells may be CSCs (7). Viperin expression was increased in CD133^+^ cells compared with CD133^–^ cells, and both CD133 and viperin expression increased when the CD133^+^ cells were serum starved. Viperin knockdown inhibited single-cell–derived spheroid formation, a functional characteristic commonly used to quantify the number of cells with self-renewal properties. The authors also used a Hoechst dye release assay to quantify the number of cells that pumped out the dye (termed the “side population” [SP]). Depleting viperin caused a decrease in the number of cells in the SP, indicating that viperin expression enhanced drug efflux, a mechanism known to promote chemotherapy resistance (7, 22). In line with these observations, MKN28 cells isolated from either spheroids or the SP had increased expression of viperin and lipogenic enzymes (7).

To explore the role of viperin in vivo, mice were inoculated with MKN28 control or viperin-knockdown cells isolated from either the SP or from single-cell–derived spheroids. Mice injected with viperin-knockdown cells developed smaller tumors that had lower levels of CD44 than did those injected with control cells. Importantly, Choi, Kim, and colleagues demonstrated that increased expression of viperin was associated with the presence of CSCs, which promote tumor initiation (7).

## Future considerations

Choi, Kim, and co-authors demonstrated that the IFN-regulated protein viperin plays two important roles in cancer progression: (a) regulating metabolic reprogramming by activating glycolysis and lipogenesis and (b) enhancing stem-like properties in CSCs (7). These observations set the stage to explore the role of ISG products in cancer cell signaling and tumorigenesis.

Not all gastric, lung, and breast cancer cell lines analyzed in this work had detectable levels of viperin. Cancer cells such as MKN45, with an undetectable level of viperin protein expression, also had a weaker induction of viperin following IFN-γ stimulation or serum starvation. It would be interesting to determine what controls the basal level of viperin expression in cancer cells with the following questions in mind: (a) Do the basal levels of viperin correlate with the basal levels of HIF-1α expression? (b) Do the relative levels of HIF-1α and viperin induction correlate with one another following serum starvation? Since previous studies have shown that HRE methylation prevents HIF-1α–dependent gene expression (23), it would be interesting to determine whether the methylation status of the viperin HRE in each of the different cell lines plays a role in viperin expression.

The expression of viperin was prompted within the TME via oxygen and fatty acid deprivation or exposure to IFN-γ (7). Multiple insults occur simultaneously in the TME, and the crosstalk between signaling cascades merits further study. For instance, a negative feedback loop involving the regulation of HIF-1α via an IFN-induced posttranslational modification, ISGylation, has been proposed to decrease HIF-1α–mediated gene expression and tumorigenesis (24). Additionally, the role of other molecular drivers should be explored. For example, HIF-2α has an established role as an mTORC1 activator (25), and its expression has been reported to be mTOR dependent (26), suggesting that HIF-2α may also play a role in viperin expression.

Most of the cancer types studied by Choi, Kim, and colleagues showed a correlation between high viperin levels and poor survival rates, except for patients with melanoma, for whom viperin levels were not directly correlated to poor survival rates (7). In addition to the link between high viperin expression and worse patient outcomes, both hypoxia/HIF-1α and CSCs have been implicated in resistance to chemotherapy, which leads to cancer progression or recurrence (27). Notably, CSCs have a high drug efflux rate, and cells expressing high levels of viperin released more Hoescht dye than did their counterparts. Taken together, the role of viperin expression in resistance to chemotherapy should be directly tested. Furthermore, IFNs play a critical role in activating the anticancer immune response (28), but metabolic reprogramming of cancer cells affects antigen presentation and recognition by immune cells (29). Thus, further studies are warranted to determine how viperin may alter immune infiltration in the TME. Given the demonstrated role for viperin in cancer cell proliferation, growth, and survival, further studies are necessary to ascertain whether viperin can be targeted to provide a clinical benefit for patients with cancer.

## Version 1 12/15/2022

Electronic publication
